# Aspirin for the older person: report of a meeting at the Royal Society of Medicine, London, 3rd November 2011

**DOI:** 10.3332/ecancer.2012.245

**Published:** 2012-02-28

**Authors:** J Armitage, J Cuzick, P Elwood, M Longley, A Perkins, K Spencer, H Turner, S Porch, S Lyness, J Kennedy, GN Henderson

**Affiliations:** 1 Professor of Clinical Trials and Epidemiology, Clinical Trials Surveillance Unit, Oxford; 2 Professor of Epidemiology. Cancer Research UK; 3 Director of Primary Care and Public Health, University of Cardiff; 4 Director, Welsh Institute for Health and Social Care, and Professor of Applied Health Policy, University of Glamorgan; 5 Professor of Radiological and Imaging Sciences, University of Nottingham Queen’s Medical Centre; 6 Director of Special Projects, europacolon; 7 Fellow, Royal Society for Public Health; 8 Director of Services, Bowel Cancer UK; 9 Executive Director of Policy and Information, Cancer Research UK; 10 Director of Operations, europacolon; 11 Executive Director, Aspirin Foundation, PO Box 223, Haslemere, GU27 3ZJ, United Kingdom

## Abstract

On November 23rd 2011, the Aspirin Foundation held a meeting at the Royal Society of Medicine in London to review current thinking on the potential role of aspirin in preventing cardiovascular disease and reducing the risk of cancer in older people. The meeting was supported by Bayer Pharma AG and Novacyl.

Professor Peter Elwood, Honorary Professor in Primary Care and Director of Primary Care and Public Health, University of Cardiff, was one of the investigators in the first trial of aspirin for the prevention of cardiovascular events [1]. His interest in the role of aspirin in cancer prevention was sparked by a botanist colleague, who pointed out that plants express salicylic acid in response to cancer cells. Why has not this phenomenon been explored in humans, the botanist asked? Since that time, many observational studies have reported an association between aspirin intake and reduced cancer risk but, in 2010, convincing evidence from long-term follow-up from prospective randomised trials showed that aspirin reduced the risk of death from cancer, and from colorectal cancer in particular [2,3]. Most recently, the CAPP2 trial demonstrated that aspirin reduces the incidence of cancer in people with Lynch syndrome, who are at increased risk of bowel cancer [4]. Despite some reservations, Professor Elwood said, there is now no reasonable doubt about the preventative effects of aspirin.

Society has a poor record of adopting risk reduction strategies. For example, US studies show that the five healthy behaviours (exercise, healthy diet, maintain low body weight, no smoking, modest alcohol use) can reduce heart disease by 80–85 per cent but adherence to these lifestyle measures (in the context of the trials) was only 3–4 per cent [5,6]. In Wales, the 1980 Caerphilly Cohort Study followed up 2,500 men for 30 years and demonstrated progressive reductions in the risks of death, vascular disease and diabetes as more protective lifestyle measures were adopted – but only 1.5 per cent of men adhered to all five healthy behaviours [7].

People do not adopt preventive measures which bring great benefit to the population because they offer little to each individual [8]. Taking aspirin daily is, by contrast, a simple measure and there is a risk that it could be perceived as a substitute for healthy behaviours because it is an easier option. In Wales, 36 per cent of the over-50s already take regular aspirin. Professor Elwood suggested that the public has acted, leaving health professionals and health authorities behind.

The question now facing society is, Who has responsibility for ensuring the appropriate use of aspirin? The treatment of disease has been delegated to health professionals, Professor Elwood said. His belief is that individuals should be given valid evidence on the balance of risks and benefits, and decide for themselves whether to take aspirin.

## Figures and Tables

**Figure 1:**
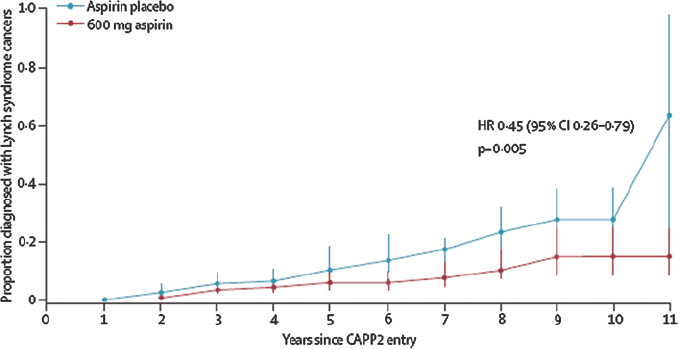
Delayed reduction in the risk of colorectal cancer in the CAPP2 trial [8]. Reproduced with kind permission from The Lancet.
