# Supplementary material for: Aspirin for the older person: report of a meeting at the Royal Society of Medicine, London, 3rd November 2011
Source: Ecancermedicalscience. 2012 Feb 28;6:245. doi: 10.3332/ecancer.2012.245 (PMC3298410; doi:10.3332/ecancer.2012.245)
Supplement: Related texts

# Sub article: Update on aspirin and cancer prevention

**Published:** 2012-02-28

**Authors:** J Cuzick

In 2009, an international consensus statement on the role of aspirin and NSAIDs for cancer prevention concluded that there was clear evidence of a chemopreventive effect on colorectal cancer and probably other cancer types but there were insufficient data on the risk-benefit profile; as a result, no definitive recommendations were made-(Professor Jack Cuzick, Professor of Epidemiology at Cancer Research UK) [1].

And lead author of that paper, said that much new evidence has since been published; the consensus statement is being updated and will be published in 2012.

Professor Cuzick described recent evidence from prospective trials that aspirin reduced cancer mortality as very important. This analysis, published in 2010, involved approximately 25,000 people and identified about 600 deaths [2,3]. It showed that daily aspirin reduced the risk of cancer mortality by 21 per cent - but the effect was seen only after a delay of 5 years. The biggest effect was on gastrointestinal cancers, with potential effects on cancers at other sites. Although there are concerns that some trials had been inappropriately excluded, this analysis provides strong evidence.

The first evidence that NSAIDs might protect against colorectal cancer was a 1983 case series in which sulindac reduced the incidence of colorectal polyps in individuals with Gardner’s syndrome [4]. Twenty years later the first randomised trial demonstrated that aspirin reduced by 35 per cent the risk of colorectal adenoma in patients with a history of colorectal cancer although only after 5 years’ use [5]. Case control and cohort studies have consistently shown a reduction in the risk of colorectal cancer associated with aspirin use [6]. Taking aspirin after a diagnosis of colorectal cancer is associated with a lower cancer-specific and overall mortality [7] but this is a relatively rapid effect and therefore probably due to another mechanism, such as inhibition of COX-2 in tumours over-expressing this enzyme. In the CAPP2 trial, daily aspirin reduced the incidence of colorectal cancer by 37 per cent overall in people with Lynch syndrome, and by 60 per cent in those who adhered to treatment for at least 2 years [8]. Again, this benefit was evident only after 5 years (Figure 1) – unusual in a high-risk population such as this, Professor Cuzick commented, contrasting it with the immediate benefit of anti-hormone therapy in women with breast cancer.

Observational studies have also found that that regular aspirin use was associated with 10–30 per cent reductions in the risk of cancers of the oesophagus, stomach and breast, and probably a lesser effect on the risk of cancers of the lung and prostate. The Nurse’s Health Study further showed that current aspirin use is associated with a 40–60 per cent lower risk of breast cancer recurrence [9]. There is less certainty about the effects of aspirin in reducing the risk of other cancers, with conflicting data for ovarian cancer and no evidence of a benefit in pancreatic cancer.

Professor Cuzick noted that several questions about aspirin remain unanswered. Which is the appropriate dose – 75 mg/day or 300 mg/day? Prospective studies [2,3] did not find a dose-response effect, he said, though alternate-day dosing had been excluded from this analysis. Studies are needed to address this question. Is it sufficient to take aspirin for 5 years and then stop, or should we continue? Which are the best ages to start and stop aspirin? It appears that gastrointestinal bleeding is a serious risk predominantly in the over-70s - is 65–70 therefore the age to stop? There is growing evidence that many patients with gastrointestinal bleeding are *Helicobacter pylori* positive: should individuals be tested and treated before starting aspirin? How important are genetic factors? Much of the evidence of the benefits of aspirin come from studies in men whereas the benefits in women have been inconsistent. This was due to negative results from the Women’s Health Study and longer follow-up of this cohort is awaited

## References

[1] Cuzick J, Otto F, Baron JA, Brown PH, Burn J, Greenwald P, Jankowski J, La Vecchia C, Meyskens F, Senn HJ, Thun M (2009). Aspirin and non-steroidal anti-inflammatory drugs for cancer prevention: an international consensus statement. Lancet Oncol.

[2] Rothwell PM, Fowkes FG, Belch JF, Ogawa H, Warlow CP, Meade TW (2011). Effect of daily aspirin on long-term risk of death due to cancer: analysis of individual patient data from randomised trials. Lancet.

[3] Rothwell PM, Wilson M, Elwin CE, Norrving B, Algra A, Warlow CP, Meade TW (2010). Long-term effect of aspirin on colorectal cancer incidence and mortality: 20-year follow-up of five randomised trials. Lancet.

[4] Waddell WR, Loughry RW (1983). Sulindac for polyposis of the colon. J Surg Oncol.

[5] Sandler RS, Halabi S, Baron JA, Budinger S, Paskett E, Keresztes R, Petrelli N, Pipas JM, Karp DD, Loprinzi CL, Steinbach G, Schilsky R (2003). A randomized trial of aspirin to prevent colorectal adenomas in patients with previous colorectal cancer. N Engl J Med.

[6] Zell JA, Ziogas A, Bernstein L, Clarke CA, Deapen D, Largent JA, Neuhausen SL, Stram DO, Ursin G, Anton-Culver H (2009). Nonsteroidal anti-inflammatory drugs: effects on mortality after colorectal cancer diagnosis. Cancer.

[7] Chan AT, Ogino S, Fuchs CS (2009). Aspirin use and survival after diagnosis of colorectal cancer. JAMA.

[8] Burn J, Gerdes AM, Macrae F, Mecklin JP, Moeslein G, Olschwang S, Eccles D, Evans DG, Maher ER, Bertario L, Bisgaard ML, Dunlop MG, Ho JW, Hodgson SV, Lindblom A, Lubinski J, Morrison PJ, Murday V, Ramesar R, Side L, Scott RJ, Thomas HJ, Vasen HF, Barker G, Crawford G, Elliott F, Movahedi M, Pylvanainen K, Wijnen JT, Fodde R, Lynch HT, Mathers JC, Bishop DT, CAPP2 Investigators (2011). Long-term effect of aspirin on cancer risk in carriers of hereditary colorectal cancer: an analysis from the CAPP2 randomised controlled trial. Lancet.

[9] Holmes MD, Chen WY, Li L, Hertzmark E, Spiegelman D, Hankinson SE (2010). Aspirin intake and survival after breast cancer. J Clin Oncol.

# Sub article: Aspirin and cancer – the views of the patients

**Published:** 2012-02-28

**Authors:** K Spencer

Europacolon (www.europacolon.com) is a pan-European patient advocacy organisation founded in 2005. It aims to unite patients, carers, healthcare professionals, politicians, the media and the public in the fight against colorectal cancer. Its goals are, explained Keith Spencer, Director of Special Projects: to create a European colorectal cancer community; to ensure formal population screening and availability, improved choice and equitable access to best treatment and care throughout Europe; and to ensure the continued implementation of conformity to EU recommendations, guidelines and policy. Patient advocacy operates at two levels, promoting awareness among the public and people with colorectal cancer, and lobbying to influence policy makers and health professionals.

Mr Spencer said the public is aware that interesting claims are being made that aspirin may reduce cancer risk and prolong survival. Where will they get their information about it? GPs seem more concerned by the possible risks of aspirin than its potential benefits and they are not particularly well informed about recent evidence. Media coverage is transitory and usually adopts a sensational perspective angle, emphasising headlines at the expense of detail. The Internet, of course, is an easy way to do research but it is full of anecdotal comment. It has an excess of information, much of which is contradictory, not easily understandable and possibly even frightening. It is easy to come away with the impression that taking aspirin is dangerous, Mr Spencer noted.

The public generally do not know enough about the risks and benefits of aspirin, or they do not understand the issues, he continued. Relevant studies should be undertaken as soon as possible to determine the effective but safe dose. If the evidence continues to support the current balance of risk and benefit, the public should be educated about a personal health strategy. Information should be targeted at key groups of people, patients who might benefit from aspirin and health professionals. It is essential not to go over people’s heads. This information should be precise and categorical about the benefits and risks such as adverse effects.

Is aspirin a low-cost lifesaver, Mr Spencer asked? If it is, it will have substantial impact on health at a low cost compared with current spending on cancer prevention and treatment, and its associated social and economic costs. It is clearly a long-term strategy, he said, and not without risk. europacolon, as the voice of the patient, wants to make a start now on collating the evidence and presenting it to the public and health professionals on a massive scale. If this does not happen, confusion and uncertainty will persist.

# Sub article: Discussion

**Published:** 2012-02-28

**Authors:** S Lyness, S Porch, M Longley, J Armitage, J Cuzick

Ms Sarah Lyness, Executive Director of Policy and Information, Cancer Research UK, described a survey in which members of the public were asked their views about taking a daily tablet to reduce cancer risk. They were open to the idea, she said, and were incredulous when they were told the tablet concerned was aspirin. They asked why these benefits were only becoming known now and they wanted to see evidence to substantiate the claimed benefit. People were more comfortable if aspirin use was supported by a strong endorser such as the Department of Health, Bayer, NICE and particularly Cancer Research UK. Most respondents were familiar with the adverse effects of aspirin and were concerned about them. Many said they would ask their doctor for information rather than a community pharmacist. GPs, on the other hand, are unfamiliar with the evidence for reducing cancer risk and want endorsement from NICE before supporting aspirin use.

Ms Sarah Porch, Director of Services at Bowel Cancer UK, agreed that people want the reassurance of an authoritative endorsement. She said that Bowel Cancer UK is aware of the confusion about aspirin from monitoring social media – the public didn’t understand the CAPP2 study, for example. There should be a single clear message from all organisations and it should be emphasised that a daily aspirin is not a substitute for a healthy lifestyle.

Professor Marcus Longley, Director, Welsh Institute for Health and Social Care, and Professor of Applied Health Policy, University of Glamorgan, pointed out that a healthy lifestyle carries no risk whereas aspirin can rarely cause stroke. Professor Cuzick said that the problems associated with aspirin occur early but its benefits come late. The public’s attention span is short and the message will have to be continually reinforced to encourage long term adherence.

Ms Porch said the public are used to making judgements about risk and benefit but they need clear information about the level of risk with aspirin. Professor Jane Armitage, Professor of Clinical Trials and Epidemiology at Oxford’s Clinical Trials Surveillance Unit, said the evidence is overwhelming for aspirin as secondary prevention of cardiovascular events but there is a lack of consensus about its role in cancer prevention. Professor Cuzick replied that there is a great deal of evidence but it needs to be synthesised and debated.

# Sub article: Where do the tablets go? Visualisation of the site of release and dispersion in the GI tract

**Published:** 2012-02-28

**Authors:** A Perkins

Tablets are the dose form everyone prefers but swallowing them is, for many patients, an unrecognised problem, said Professor Alan Perkins, Professor of Radiological and Imaging Sciences, University of Nottingham Queen’s Medical Centre.

It is known that children and some patient groups (eg, those with stroke, heart failure or diabetes, and frail elderly people) have difficulty swallowing tablets but a US study found that 40 per cent of adults had difficulty swallowing tablets and the problem was more common among women and men [1].

The way a dose form is taken has a strong influence on its transit time from the mouth to the stomach – shorter when sitting upright and if the dose is swallowed with water – and may influence its therapeutic effect. For example, the rate of onset of effect of aspirin is more rapid if a tablet is chewed than if it is swallowed in solution or as a whole tablet [2]. In healthy volunteers, the mean time to 50 per cent inhibition of thromboxane synthesis was 5.0 minutes after chewing, 7.6 minutes after the solution and 12.0 minutes after a solid tablet (Figure 1[2]). Surprisingly, morbidities which affect posture, such as kyphosis, do not appear to affect transit time.

Tablet shape and coating are also important. Transit time is shorter for oblong tablets than round ones, and for film-coated tablets compared with uncoated tablets [3]. Drug dispersion, and therefore absorption, is influenced by the disintegration characteristics of a tablet after administration. Generic tablets from different manufacturers may have different disintegration characteristics from the original brand and from one another.

Imaging studies have shown that a dose unit can remain lodged in the oesophagus for 60 minutes after ingestion but the individual may be unaware of this (Figure 2). Incomplete swallowing leading to an increased local concentration of drug can cause significant mucosal damage, most frequently affecting the lower oesophagus [4,5]. This is a significant risk for patients taking a bisphosphonate because these drugs have been associated with severe oesophageal reactions. Other drugs associated with erosive oesophageal reactions include some antibiotics (doxycycline, clindamycin, trimethoprim), NSAIDs, ferrous sulphate, theophylline and zidovudine.

Professor Perkins described a study using γ scintigraphy to quantify the oesophageal transit times of a film-coated tablet and gelatin capsule formulation of the bisphosphonate etidronate in 25 healthy volunteers. This technique is superior to a barium swallow, which is unphysiological, and to MRI because this poses practical difficulties in positioning the patient. Using a radio-labelled tablet, he showed that the transit time from the mouth to the stomach was significantly longer for the capsule (mean 24 seconds) than the tablet (mean 3.3 seconds [6]).

The formulation can be manipulated to target tablet disintegration and drug release at specific sites in the gastrointestinal tract to reduce the risk of gastrointestinal bleeding – for example by enteric coating or pH-controlled release. γ scintigraphy can be used to provide visual confirmation of the site at which the drug is released and this can be correlated with blood levels.

## Figures and Tables

**Figure 1:** Time to 50% inhibition of thromboxane synthesis after ingestion of aspirin by chewing a tablet, swallowing a solution or swallowing a whole tablet [2].

**Figure 2:** γ scintigraphy showing gelatin capsule lodged in the lower oesophagus for 60 minutes after ingestion.

## References

[1] Swallowing an underestimated problem in adults, says survey (www.in-pharmatechnologist.com/Materials-Formulation/Swallowing-an-underestimated-problem-in-adults-says-survey)

[2] Feldman M, Cryer B (1999). Aspirin absorption rates and platelet inhibition times with 325-mg buffered aspirin tablets (chewed or swallowed intact) and with buffered aspirin solution. Am J Cardiol.

[3] Perkins AC, Wilson CG, Frier M, Blackshaw PE, Dansereau RJ, Vincent RM, Wenderoth D, Hathaway S, Li Z, Spiller RC (2001). The use of scintigraphy to demonstrate the rapid esophageal transit of the oval film-coated placebo risedronate tablet compared to a round uncoated placebo tablet when administered with minimal volumes of water. Int J Pharm.

[4] Kikendall JW, Friedman AC, Oyewole MA, Fleischer D, Johnson LF (1983). Pill-induced esophageal injury. Case reports and review of the medical literature. Dig Dis Sci.

[5] Eng J, Sabanathan S (1991). Drug-induced esophagitis. Am J Gastroenterol.

[6] Perkins AC, Wilson CG, Frier M, Vincent RM, Blackshaw PE, Dansereau RJ, Juhlin KD, Bekker PJ, Spiller RC (1999). Esophageal transit of risedronate cellulose-coated tablet and gelatin capsule formulations. Int J Pharm.

# Sub article: Aspirin – risks and benefits

**Published:** 2012-02-28

**Authors:** P Elwood

Three authoritative reports have now identified personal responsibility as the key to achieving good health within current resources:

- The Wanless report Securing good health for the whole population stated ‘...health services in the UK are unsustainable in their current form unless members of the public are ‘fully engaged’ and take responsibility for their own health [1]’
- The Department of Health stressed the importance and potential of healthy living:’...the major aim is to ‘empower’ the ‘millions of individuals… to make the right choice’ as a means to reduce the strain on public services [2]
- The King’s Fund concluded that ‘the NHS should provide information, advice and support ‘to enable everyone to prevent illness and lead healthier lives [3]’

Professor Elwood said there is no disagreement about the value of aspirin as secondary prevention of cardiovascular events but there is a debate about its role in healthy elderly people, in whom the balance of risk (bleeding events) and benefits (fewer cardiovascular events) may be less favourable. Weighing the pros and cons is not straightforward. It may not always be possible to recognise cardiovascular disease when its first manifestation is a fatal event; and a reduced risk of cancer should be added to the benefits side of the equation. Bleeding events are not all the same. Aspirin increases the risk of gastric bleeding by about 60 per cent – equivalent to 2–3 cases per 1,000 users – but Professor Elwood challenged the assumption that they should all be classed serious events: a haemorrhagic stroke is much more serious than a gastric bleeding event and is much rarer (about 2–3 per 10,000).

In six primary prevention trials (albeit not including very elderly people), aspirin was associated with an excess of bleeding events but mortality was similar to that among people taking placebo [4,5]. In these studies, the incidence of bleeding events was 2.2/1,000/year among aspirin users and 1.3/1,000/year among those assigned to placebo. The case fatality of those with bleeding events was 1.8 and 3.1 per cent respectively. The incidence of deaths from bleeding events was 0.040/1,000/year with aspirin and 0.049/1,000/year with placebo [4].

Any spontaneous bleeding event that occurs in someone taking aspirin will be blamed on the drug, Professor Elwood commented. Such misattribution influences the views of GPs, who are very risk averse in their prescribing. Individuals should be given relevant information and allowed to decide for themselves whether the balance of risk and benefit was favourable, he said.

It is unfortunate that no steps were taken in the large aspirin trials to evaluate ways to reduce bleeding risk. Simple measures could reduce the risk, such as taking aspirin at night (the greatest reduction in myocardial infarction risk occurs in the morning [6], gastric repair proteins are highest at night [7,8]), eradication of *H pylori* [9], treatment with a proton pump inhibitor [10,11], and taking aspirin with a source of calcium [12,13] (such as a glass of milk).

People contemplating taking low-dose aspirin should be given information about the risks. This includes the importance of checking blood pressure due to the small increased risk of a haemorrhagic stroke (increased by about 60 per cent, or 2–3/10,000/year). There is evidence of a rebound 3-fold increase in the risk of vascular events after stopping aspirin [14–16]. Even after a gastric bleeding event, the possibility of continuing aspirin with additional treatment with a proton pump inhibitor should be considered: in one small study in patients with peptic ulcer bleeding during use of low-dose aspirin (n=156), 30-day mortality due to cardiovascular, cerebrovascular, or gastrointestinal complications was greater in those who stopped aspirin than those who continued it in combination with pantoprazole (10.3 vs 1.3 per cent) despite a higher frequency of continued peptic ulcer bleeding (10.3 vs 5.4 per cent [17]).

During the discussion, Professor Elwood agreed that age influences the degree of autonomy people want in their decision-making. Older people tend to believe that the doctor knows best whereas younger adults are prepared to be more challenging. The advice of a health professional will also remain important for people who have difficulty understanding the evidence, especially those with disabilities or who are living in disadvantaged communities.

## References

[1] Wanless D (2004). Securing good health for the whole population.

[2] Department of Health UK (2004). Choosing health: making healthier choices easier. A Public Health White Paper.

[3] King’s Fund (2004). Public Attitudes to Public Health Policy.

[4] Guise J-M, Mahon SM, Aicken M (2002). Aspirin for the prevention of cardiovascular events: a summary of the evidence. Ann Intern Med.

[5] Morgan G (2009). Aspirin for the primary prevention of vascular events?. Public Health.

[6] Ridker PM, Manson JE, Buring JE, Muller JE, Hennekens CH (1990). Circadian variation of acute myocardial infarction and the effect of low-dose aspirin in a randomized trial of physicians. Circulation.

[7] Semple JI, Newton JL, Westley BR, May FE (2001). Dramatic diurnal variation in the concentration of the human trefoil peptide TFF2 in gastric juice. Gut.

[8] Johns CE, Newton JL, Westley BR, May FE (2005). The diurnal rhythm of the cytoprotective human trefoil protein TFF2 is reduced by factors associated with gastric mucosal damage: ageing, Helicobacter pylori infection, and sleep deprivation. Am J Gastroenterol.

[9] Lanas A, Fuentes J, Benito R, Serrano P, Bajador E, Sáinz R (2002). Helicobacter pylori increases the risk of upper gastrointestinal bleeding in patients taking low-dose aspirin. Aliment Pharmacol Ther.

[10] Chan FK, Sung JJ (2001). Role of acid suppressants in prophylaxis of NSAID damage. Best Pract Res Clin Gastroenterol.

[11] Scheiman JM, Yeomans ND, Talley NJ, Vakil N, Chan FK, Tulassay Z, Rainoldi JL, Szczepanski L, Ung KA, Kleczkowski D, Ahlbom H, Naesdal J, Hawkey C (2006). Prevention of ulcers by esomeprazole in at-risk patients using non-selective NSAIDs and COX-2 inhibitors. Am J Gastroenterol.

[12] Grau MV, Baron JA, Barry EL, Sandler RS, Haile RW, Mandel JS, Cole BF (2005). Interaction of calcium supplementation and nonsteroidal anti-inflammatory drugs and the risk of colorectal adenomas. Cancer Epidemiol Biomarkers Prev.

[13] Bolland MJ, Avenell A, Baron JA, Grey A, MacLennan GS, Gamble GD, Reid IR (2010). Effect of calcium supplements on risk of myocardial infarction and cardiovascular events: meta-analysis. BMJ.

[14] Collet JP, Montalescot G, Blanchet B, Tanguy ML, Golmard JL, Choussat R, Beygui F, Payot L, Vignolles N, Metzger JP, Thomas D (2004). Impact of prior use or recent withdrawal of oral antiplatelet agents on acute coronary syndromes. Circulation.

[15] Rodríguez LA, Cea-Soriano L, Martín-Merino E, Johansson S (2011). Discontinuation of low dose aspirin and risk of myocardial infarction: case-control study in UK primary care. BMJ.

[16] Biondi-Zoccail GGl, Lotrionte M, Agostoni P, Abbate A, Fusaro M, Burzotta F, Testa L, Sheiban I, Sangiorgi G (2006). A systematic review and meta-analysis on the hazards of discontinuing is not adhering to aspirin among 50.279 patients at risk for coronary artery disease. Eur Heart J.

[17] Sung JJ, Lau JY, Ching JY, Wu JC, Lee YT, Chiu PW, Leung VK, Wong VW, Chan FK (2010). Continuation of low-dose aspirin therapy in peptic ulcer bleeding: a randomized trial. Ann Intern Med.

# Sub article: Aspirin prophylaxis and public health

**Published:** 2012-02-28

**Authors:** M Longley

There are three myths about the role that the public can play in determining their health, said Professor Marcus Longley, Director, Welsh Institute for Health and Social Care, and Professor of Applied Health Policy, University of Glamorgan: they cannot weigh risks and benefits; scientific uncertainty is dangerous for lay people; and most people would simply rather not know.

As with all myths there is an element of truth in these statements but modern thinking about public health is more nuanced. An independent report based on the latest evidence from behavioural economics and psychology concluded that ‘People do not smoke or drink too much because they are ignorant, stupid or perverse – rather, it is the combination of the enjoyment that they get from these things and wider social or other environmental factors that mean they find it hard to adopt healthier behaviours [1]’. People gain something from unhealthy behaviours and they make an implicit trade-off of risks and benefits in choosing to continue them. Government needs to take this into account when forming its public health messages, a second report found, and it should place greater weight on informed choice and individual capacity [2].

The goal of public health policy has long been to increase life expectancy but this has been pursued at the cost of quality of life in later years [3]. Recent years have shown that preaching to people does not persuade them to change their lifestyle, so why not take a medicine that achieves that end more easily? The combined oral contraceptive is a case in point. The counter argument, Professor Longley said, is that it is risky, usually does not work, it discourages people from trying to be healthy and it turns people into patients.

What do we understand by ‘risky’? The risk of fatality for a 50 year-old man taking aspirin to prevent a coronary event is 10 per 100,000 person-years of use. This is about the same risk as being a passenger in a car (11 per 100,000 person-yrs) and far lower than for a motor-cyclist (450 per 100,000 person-years [4]). The interpretation of risk varies between individuals and depends partly on what outcomes – death, disability, pain – are important to them.

Five years ago, before the association between aspirin and cancer risk reduction was publicised, Professor Longley and Professor Elwood convened a Citizens’ Jury to consider what information should be provided to the public and how it should be delivered [5]. Citizens’ Juries are not a new idea, he said, because they have been determining questions of guilt and innocence for over 800 years. In this case, they are based on the premise that ordinary people – if given the opportunity and enough time, support, resources – are capable of arriving at decisions about complex policy matters. Paid jurors consider, over 3–4 days, evidence from expert witnesses whom they cross-examine; after facilitated discussion, they jointly formulate policy recommendations.

In this trial, the 16-member jury considered the case of cardioprevention with low dose aspirin. Jury members were a diverse group with contrasting views pre-trial views about aspirin but they collaborated effectively to develop 10 recommendations (Table 1). These show that jurors were less protective of themselves than is believed. It is possible to have an adult conversation about health with the general public, Professor Longley commented.

Considering the three myths, Professor Longley said that the Citizen’s jury showed that the public can weigh scientific evidence, though they need a process and support to do so and this is resource-intensive. They can deal with uncertainty – jurors wanted to be informed even about issues that were controversial among scientists. It was less clear whether the jury refuted the assertion that people would rather not know.; Professor Longley suspected that health was not an important issue for some people.

Questions remain. How can the views of vulnerable people be represented? What are the resource implications for clinicians? Can this approach work for issues that are less clear than the aspirin debate? Do health professionals and the public share a common understanding about what constitutes ‘health’, and is it more important to some people than others?

## Figures and Tables

**Table 1:** Citizen’s jury recommendations on the use of low-dose aspirin for cardioprevention [5].

## References

[1] Mulgan G (2010). Influencing public behaviour to improve health and wellbeing.

[2] Reeves R (2010). A liberal dose? Health and wellbeing - the role of the state.

[3] Bernstein H, Cosford P, Williams A (2010). Enabling effective delivery of health and wellbeing.

[4] Cohen JT, Neumann PJ (2007). What’s more dangerous, your aspirin or your car? Thinking rationally about drug risks (and benefits). Health Aff (Millwood).

[5] Elwood P, Longley M (2010). My health: whose responsibility? A jury decides. J Epidemiol Community Health.

# Sub article: Aspirin and cardiovascular disease

**Published:** 2012-02-28

**Authors:** J Armitage

There is good evidence to support the use of aspirin as secondary prevention of cardiovascular events but not for primary prevention in healthy people at low risk. The case for primary prevention in people at high risk (eg the elderly, those with diabetes or multiple risk factors) is unclear. Unsurprisingly, the public is confused by positive and negative headlines about aspirin, acknowledged Professor Jane Armitage, Professor of Clinical Trials and Epidemiology, Clinical Trials Surveillance Unit, Oxford.

Summarising the evidence for secondary prevention, she said the Antithrombotic Trialists’ Collaboration had shown, using patient-level data, that antiplatelet treatment reduced major coronary events and ischaemic each by one-third and vascular mortality by one-sixth [1]. These benefits greatly outweigh the risks from bleeding, she said.

There are two possible strategies for avoiding a first vascular event in individuals at low-risk: start aspirin immediately and continue for life, or delay aspirin until definite signs or symptoms of vascular disease emerge. Immediate treatment may avoid first heart attacks and strokes but exposes users to an increased risk of cerebral haemorrhage and gastrointestinal bleeding, for many years.

The assessment of the potential benefit of aspirin must take into account the changing background incidence of vascular events and alternative interventions. Vascular mortality has declined substantially in many countries among younger adults and both vascular and coronary heart disease among 70–79 year-olds (Figure 1) (data for the over-80s are not sufficiently reliable). Furthermore, primary prevention through treatment with statins and reduction of blood pressure can halve the risk of vascular events, including coronary events, and the risk of stroke.

There have been six large studies of primary prevention with aspirin, with approximately 660,000 person-years of follow-up in a population of 95,000 largely healthy people, of whom about one-third were over 65 years old [1]. The risks of stroke and vascular death were not significantly reduced; the risk of coronary heart disease was significantly reduced by 18 per cent but there was no reduction in coronary deaths; combining these endpoints, the overall risk reduction of 12 per cent was statistically significant (Figure 2). Against this, the overall risk of gastrointestinal or other extracranial bleeding event was increased by 54 per cent (Figure 3), although it should be noted that data on bleeding events were not collected with care. Importantly, the risk of bleeding correlated with the risk of coronary events, suggesting that people at greatest risk from heart disease were also at increased risk from the adverse effects of aspirin.

Current management guidelines recommend life-long aspirin to all those whose 10-year risk of coronary heart disease is predicted to be 10–20 per cent. However, this recommendation assumes that bleeding risk remains approximately constant (about 0.1%/year) for everyone or is determined only by age. However, the risk of bleeding is also increased by comorbidity (such as diabetes), smoking, raised blood pressure and being a man. There are, therefore, several factors that can increase the risk of bleeding events. It is also evident that the absolute benefits of primary prevention with aspirin are greatly reduced when given with other drugs which halve the risk coronary events in individuals at moderate or high risk (Figure 4).

Professor Armitage summarised the arguments by saying that primary prevention with statins and anti-hypertensive drugs can halve the risk of vascular events. The benefits of adding aspirin to this strategy are small and are associated with small risks of bleeding. It is therefore not appropriate to recommend aspirin for a large proportion of the general population. On the other hand, there are sufficient grounds for individuals to make a rational choice to take aspirin if they consider the benefits to be worth the risks for themselves. Studies now underway will provide evidence in important subgroups such as people with diabetes and older people in coming years.

## Figures and Tables

**Figure 1[1]:** Mortality trends for vascular disease (left) and CHD (right) aged 70–79.

**Figure 2[1]:** RCTs of aspirin for primary prevention.

**Figure 3[1]:** Gastro-intestinal bleed (or other major extracranial bleed) in primary prevention trials.

**Figure 4:** The effect of concurrent treatment on the efficacy of primary prevention with aspirin in individuals at moderate and high risk (A=aspirin, C=controls [1]).

## References

[1] Antithrombotic Trialists’ Collaboration (2002). Collaborative meta-analysis of randomised trials of antiplatelet therapy for prevention of death, myocardial infarction, and stroke in high risk patients. BMJ.

# Sub article: Open discussion

**Published:** 2012-02-28

**Authors:** H Turner

Dr Hugh Turner, GP in County Durham and a Fellow of the Royal Society for Public Health, said that he had come to the meeting hoping to find a clear message about using aspirin that he could take home to his patients. Tongue in cheek, he summarised the conclusions of the meeting in the form of advice to one of his patients in this way. “There is good evidence that aspirin, when taken (sitting upright) every day as an oblong tablet of 75 mg–300 mg, reduces cancer risk after 5 years. Taking it at night may increase the benefit. Before starting, have a test for *H pylori*. It’s your decision whether to take it.”

Dr Turner’s point was that there is clearly room to improve the clarity of the information that patients receive about aspirin, and he added that there is also a need to educate health professionals.

In discussion, participants expressed reluctance to see aspirin replace the five healthy behaviours but it was acknowledged that most people will not make the necessary changes to their lifestyle. Public health initiatives, though they have the potential to achieve a greater risk reduction than aspirin, are not popular with the public.

# Sub article: Prophylactic aspirin – the subjects’ choice

**Published:** 2012-02-28

**Authors:** J Armitage, P Elwood, H Turner, M Smith, J Cuzick

Following the meeting, participants were joined by invited guests to discuss the implications of the day’s proceedings. It was pointed out that, back in the 1960s, it was common to prescribe aspirin to reduce the risk of metastasis in women with breast cancer. This was not supported by evidence from randomised trials and the practice fell into disuse but it would not be difficult to conduct an appropriate trial to test the hypothesis. Professor Armitage said the evidence that aspirin reduces cancer risk, though strong for colorectal and oesophageal cancer, is not universally convincing. She said most evidence comes from the UK. The studies included in the analyses had been selected (introducing the risk of bias) and were not designed to measure this endpoint. We still lack evidence for an overall reduction of cancer risk.

Professor Elwood emphasised that it is important to obtain patients’ preferences about outcomes. The impact of a gastric bleeding event is much less than that of a stroke, he said, and aspirin is not associated with an increased risk of fatal bleeding (at least in the under-65s). Cerebral haemorrhage is a true disaster for patients, he added.

Dr Turner pointed out that most bleeding events are detected on routine screening as a fall in haemoglobin; patients are usually unaware of it. Aspirin is suspended for a few days, a proton pump inhibitor is prescribed, and aspirin is reintroduced pending a decision about further investigations. He added that almost all the elderly patients in his practice are being prescribed a proton pump inhibitor, so their risk of gastric bleeding is reduced. One participant described the ‘soft side effects’ of aspirin, such as delayed surgery, not stopping bleeding after a blood sample is taken or extensive bruising after minor trauma. Such events do not appears in the statistics, she added.

GP Dr Mike Smith recalled a similar debate when the combined oral contraceptive was first introduced. The use of a drug by healthy women, when other methods of contraception were available, was initially controversial but the perceived benefits were so great that they outweighed the disadvantages. Dr Smith speculated that we might reach the same situation with aspirin if we could say to the average person, ‘these are the facts now make up your own mind’. But it would be quite complicated to describe in everyday language the risks and benefits for each individual. Professor Cuzick suggested that epidemiologists could develop a tool that enabled GPs to estimate an individual’s risk of cardiovascular disease, cancer and bleeding events. This would help GPs communicate with patients and simplify decision-making, he said. It was pointed out that, while some patients look to their GP for guidance, others want advice or even to be told what to do.
